# Effectiveness of protein supplementation combined with resistance training to counteract disproportional fat-free mass loss following metabolic bariatric surgery: rationale and design of the ENRICHED randomised controlled trial

**DOI:** 10.1136/bmjopen-2025-108346

**Published:** 2025-12-29

**Authors:** Bente M de Roos, Lin-Lin Yessica Yeh, Puck S van den Hooff, Malou A H Nuijten, Jos W R Twisk, Ronald S L Liem, Gijs J D van Acker, Johan L Severens, Tim Kambic, Mireille J M Serlie, Valerie M Monpellier, Thijs M H Eijsvogels, Maria T E Hopman, Jan H M Karregat

**Affiliations:** 1 Medical Biosciences, Radboud University Medical Center, Nijmegen, The Netherlands; 2 Endocrinology and Metabolism, Amsterdam University Medical Center, Amsterdam, The Netherlands; 3 Nederlandse Obesitas Kliniek, Huis ter Heide, The Netherlands; 4 Amsterdam Public Health research institute, Vrije Universiteit Amsterdam, Amsterdam, The Netherlands; 5 Epidemiology and Data Science, Amsterdam University Medical Center, Amsterdam, The Netherlands; 6 Department of Surgery, Groene Hart Hospital, Gouda, The Netherlands; 7 Nederlandse Obesitas Kliniek, The Hague and Gouda, The Netherlands; 8 Department of Surgery, Medical Centre Haaglanden, The Hague and Leidschendam, The Netherlands; 9 Severens HTA Consultancy, Venray, The Netherlands; 10 Medical Sciences in Sport and Exercise, University of Ljubljana, Ljubljana, Slovenia; 11 Endocrinology and Metabolism, Yale School of Medicine, New Haven, Connecticut, USA

**Keywords:** Bariatric Surgery, Obesity, Randomized Controlled Trial, Clinical Protocols

## Abstract

**Introduction:**

Metabolic bariatric surgery (MBS) can lead to substantial fat-free mass loss (FFML) due to malnutrition, decreased protein intake and insufficient physical activity. Disproportional FFML has been associated with an increased risk for adverse health outcomes. Resistance training (RT) combined with protein intake contributes to maintenance and increase of fat-free mass (FFM) in healthy individuals. However, it is unclear whether RT and protein supplementation can prevent FFML after MBS.

**Methods and analysis:**

In the EffectiveNess of pRotein supplementatIon Combined witH resistance Exercise training to counteract Disproportional fat-free mass loss following metabolic bariatric surgery (ENRICHED) randomised controlled trial, 400 patients scheduled to undergo MBS will be randomised in a 1:1 ratio to the ENRICHED perioperative care programme (intervention group) or the standard perioperative care programme of the Dutch Obesity Clinic (control group). The study is currently recruiting participants at two centres in the Netherlands: Nieuwegein and Amsterdam. The postoperative standard programme consists of 13 group sessions spread over a period of 18 months. As part of the ENRICHED programme, RT and protein supplementation will be added 3 weeks after MBS. Additional whole-body RT consists of home-based training sessions two to three times a week, and supervised RT sessions of 45–60 min once weekly, performed at 60–75% of one-repetition maximum (1-RM). Protein supplementation will start by adding 20 g of whey protein to the daily intake. The supplementation will be gradually increased with 20 g every 4 weeks until a total of 60 g whey protein a day is reached. After 12 weeks of protein supplementation, the focus shifts towards incorporating protein-rich food products into the daily dietary intake. The primary endpoint is the prevalence of disproportional FFM loss, defined as FFML/total weight loss ≥30%, at 3 months post-MBS. Secondary endpoints are differences in body composition, muscle strength and function, cardiorespiratory fitness, (cardio)metabolic health, health-related quality of life, gastrointestinal discomfort, cost-effectiveness of the intervention and treatment satisfaction. Outcomes will be assessed preoperatively and at 3, 6 and 12 months postoperatively.

**Ethics and dissemination:**

The study protocol V.2.0 was approved by the Medical Research Ethics Committee Oost-Nederland (NL-OMON57119) on 9 April 2025. All participants will provide written informed consent prior to enrolment. Study findings will be disseminated through peer-reviewed publications and conference presentations. Insights gained in this study will provide evidence for a patient-tailored intervention that could be implemented in clinical practice.

**Trial registration number:**

NCT07156552.

**STRENGTHS AND LIMITATIONS OF THIS STUDY**

- This is a large-scale, multicentre randomised controlled trial to evaluate the combined effect of resistance training and protein supplementation on fat-free mass preservation in the early postoperative phase after metabolic bariatric surgery.
- The intervention is embedded in routine clinical care, enhancing feasibility, adherence and potential implementation in clinical practice.
- Objective measurements of physical activity, body composition and dietary intake provide comprehensive outcome data.
- Blinding of participants, healthcare providers and researchers is not feasible due to the nature of the intervention, which may introduce performance bias.
- Although dual-energy X-ray absorptiometry is the gold standard for body composition assessment, it is only used in a subgroup (n=100) due to cost and feasibility constraints.

## Introduction

 Obesity has become a pressing global health challenge, with its prevalence and severity continuing to rise across populations worldwide.^1^ Metabolic–bariatric surgery (MBS) is one of the most effective long-term interventions for the treatment of severe obesity, with a total weight loss (TWL) around 30% at 12 months postsurgery.^2^ In addition to weight reduction, MBS has been shown to be effective in improving metabolic health and reducing obesity-associated complications, thereby enhancing overall health-related quality of life (HRQoL).^3 4^

Despite these benefits, weight loss following MBS comprises not only fat mass (FM) but also a considerable proportion of fat-free mass (FFM). Studies have shown that approximately 25% of the TWL within the first 3 months post-MBS consists of FFM.^5^ FFM plays a crucial role in key physiological functions, including glucose metabolism, resting energy expenditure, thermoregulation and physical capacity.^6^ In turn, disproportional loss of FFM has been associated with impaired metabolic health and functional capacity, and an increased risk for chronic diseases ultimately leading to lower HRQoL.^47^,^9^ In the present study, we define a proportion of FFM loss (FFML) ≥30% as disproportional, based on previous evidence linking this ratio of FFML/TWL to an increased risk of major adverse cardiovascular events and mortality.^10^ The insights in the health consequences of FFML have shifted treatment goals in obesity treatment, coming from maximising weight loss to improving body composition and thereby general health.^11^

Resistance training (RT) complemented with protein supplementation has been shown to be effective for maintaining and improving FFM in healthy^12^ and clinical populations.^13^ However, patients post-MBS often fail to meet the daily recommended protein intake of 60 g/day^14^ and physical activity (PA) levels of 150 min of moderate-intensity PA.^15 16^ Evidence of the effectiveness of RT and protein supplementation in the early postoperative phase (3–12 months) following MBS remains limited. Previous studies have been rather small and involved homogeneous populations,^17^ or have focused solely on the late postoperative period (2–7 years post-MBS), with inconsistent findings.^18 19^

The ‘EffectiveNess of pRotein supplementatIon Combined witH resistance Exercise training to counteract Disproportional fat-free mass loss following metabolic bariatric surgery (ENRICHED)’ study aims to evaluate the effectiveness of RT complemented with protein supplementation in the standard perioperative care programme to counteract disproportional FFML in the first 3–12 months following MBS. Additionally, the effect of the intervention on body composition, (cardio)metabolic health, muscle strength and function, cardiorespiratory fitness, HRQoL, dietary protein intake and habitual PA patterns will be evaluated.

## Methods and analysis

### Study design

**Figure 1:**
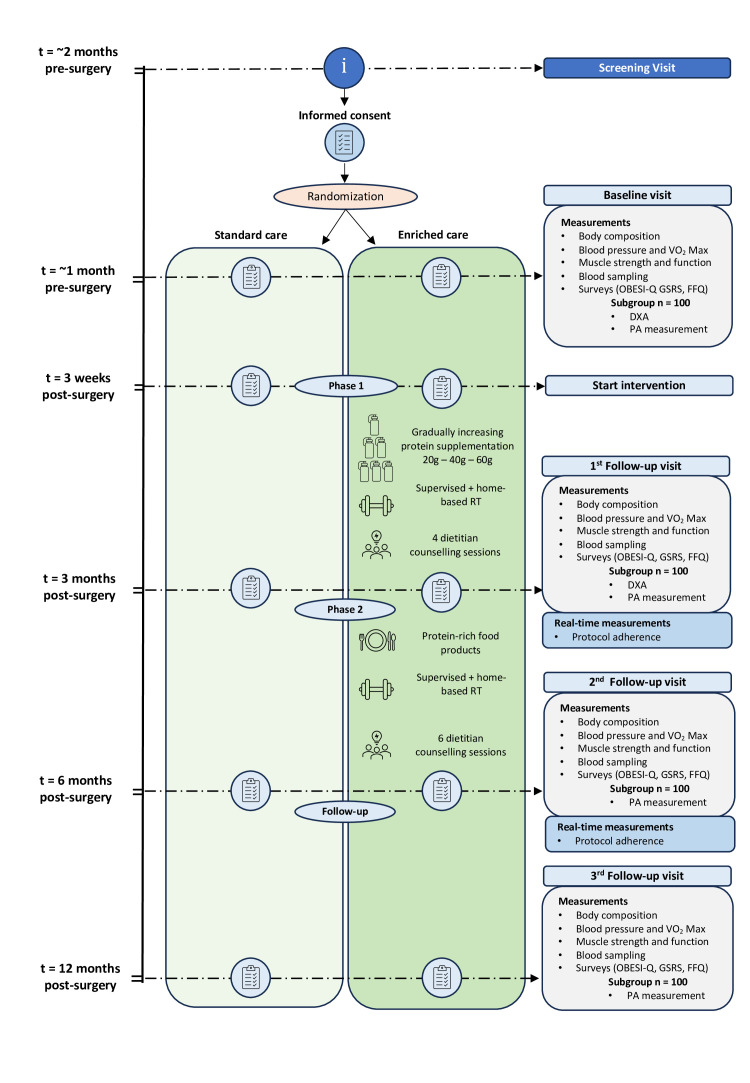
Schematic overview of the ENRICHED trial timeline and study procedures. DXA, dual-energy X-ray absorptiometry; ENRICHED, EffectiveNess of pRotein supplementatIon Combined witH resistance Exercise training to counteract Disproportional fat-free mass loss following metabolic–bariatric surgery; FFQ, Food Frequency Questionnaires; g, gram; GSRS, Gastrointestinal Symptom Rating Scale; OBESI-Q, a validated patient reported quality of life measure for patients with obesity PA, physical activity; RT, resistance training; VO_2_ Max, maximum uptake of oxygen.

The ENRICHED study is a multicentre randomised controlled trial (RCT) with two parallel arms evaluating the effectiveness of a combined intervention of RT and protein supplementation (figure 1).

### Study setting and recruitment

This study is currently being conducted at two centres of the Nederlandse Obesitas Kliniek (NOK, Dutch Obesity Clinic), located in Nieuwegein and Amsterdam, in the Netherlands. The NOK consists of a national network of 10 sites throughout the Netherlands which provide a perioperative care programme for patients undergoing MBS. Patients qualify for the perioperative care programme in accordance with the International Federation for the Surgery of Obesity and Metabolic Disorders criteria; body mass index (BMI) ≥40 kg/m^2^ or BMI ≥35 kg/m^2^ in combination with obesity associated diseases (eg, type 2 diabetes or hypertension).^20^ Both Roux-en-Y gastric bypass (RYGB) and sleeve gastrectomy (SG) procedures are included in this trial to enhance generalisability of our findings and to allow examining whether the intervention is effective across both surgical techniques.

Recruitment of participants began in May 2025 and is expected to continue until December 2026. Patients are verbally informed about the study by the treating healthcare professional during the intake consultation at the participating centres. If patients provide their permission to be contacted for further information, a member of the research team evaluates eligibility via the electronic health record and contacts the patients to discuss potential participation. Inclusion and exclusion criteria are summarised in table 1.

**Table 1:** Inclusion and exclusion criteria

Inclusion criteria	Exclusion criteria
Scheduled to undergo RYGB or SG in one of the participating centres.	Allergic or sensitive to milk proteins, or lactose intolerant.
Age ≥18.	Diagnosed renal insufficiency (ie, eGFR <30 mL/min).
Able to understand and perform the study procedures.	Diagnosed intestinal diseases influencing uptake of protein (ie, active inflammatory bowel disease, Crohn’s disease).
	Inability to perform any resistance exercises (eg, severe physical limitations).
	Previous metabolic–bariatric surgery.
	Presence of medical devices (ie, pacemaker, neurostimulator) which are contraindicated for bioelectrical impedance analysis.

eGFR, estimated glomerular filtration rate; RYGB, Roux-en-Y gastric bypass; SG, sleeve gastrectomy.

### Randomisation, blinding and treatment allocation

A total of 400 patients will be enrolled and randomly assigned in a 1:1 ratio to either the intervention or control group. Randomisation will be stratified by sex to ensure a balanced sample across both groups, which does not imply equal representation of men and women. A computerised randomisation system (Castor Electronic Data Capture (EDC), Castor, Amsterdam, the Netherlands) will be used with variable block sizes of 16 and 24 to ensure allocation concealment. A subgroup of 100 patients will undergo additional measurements, including dual-energy X-ray absorptiometry (DXA) for body composition assessment and objective PA measurements using thigh-worn accelerometry. Blinding of the patients, involved healthcare professionals and outcome assessors is not possible due to the nature of the intervention.

### Standard care

The standard perioperative care programme is focused on supporting patients in adopting a new healthy lifestyle incorporating mental and physical health. The objectives of the programme are to provide guidance to enhance adherence to healthy lifestyle and behaviour changes, ensure understanding of the benefits of a healthy lifestyle and improve both physical and mental health. The programme is provided by a multidisciplinary team consisting of bariatric physicians, dietitians, psychologists, kinesiologists, surgeons and internists. The programme consists of group sessions with up to 10 patients per group; each group is assigned to one of the healthcare professionals who functions as coach. Preoperatively, patients participate in group sessions on a weekly basis for 4 weeks. Postoperatively, the group sessions are repeated every 3–6 weeks for a 15-month period. In addition, medical checks are performed by a bariatric physician at 3 weeks postsurgery and repeated every 3 months, up to 1 year post-MBS.

Allocation to our control group does not restrict participants from engaging in RT or consuming additional protein. This is emphasised by the research team during recruitment. Moreover, the research team is trained to ensure equipoise delivery during recruitment by presenting both study arms as equally important for the aim of this study.

### Intervention

The ENRICHED intervention consists of two consecutive phases, starting 3 weeks after surgery and continuing for a total of 24 weeks (figure 1). Phase 1 (3–14 weeks post-MBS) focuses on home-based and supervised RT and gradually increasing protein supplementation. Phase 2 (15–26 weeks post-MBS) continues with the same RT protocol while transitioning from protein supplementation to enhancing dietary protein intake.

#### Resistance training

##### Standard care

As part of the standard perioperative care programme, patients are provided with a semistructured home-based RT protocol, which can be performed using little to no equipment. This home-based RT protocol includes three difficulty levels with six different whole-body RT exercises. Patients are advised to perform 2–3 weekly RT sessions on non-consecutive days. Each exercise is completed after three sets of 6–12 repetitions, with 60 s of rest between sets. Patients are advised to progress to a higher difficulty level once they are able to perform three sets of 12 repetitions at the current difficulty level.

##### Intervention: home-based and supervised RT

Patients in the intervention group will receive an adapted version of the home-based RT protocol that is part of the standard care. This adapted protocol includes four difficulty levels with six adjusted whole-body RT exercises to ensure activation of all major muscle groups (online supplemental table 1). In the intervention protocol, each exercise is completed after four sets of 8–12 repetitions, with 60 s of rest between sets. All exercises in the home-based RT protocol can be performed using no to little equipment to ensure an easy translation to the home environment.

As part of the intervention, weekly RT sessions are provided at the clinic and are supervised by an experienced kinesiologist. The supervised RT protocol includes four machine-based whole-body exercises and one exercise using body weight (online supplemental table 2A). During the first supervised session, the kinesiologist will guide patients through the home-based RT protocol to ensure they understand how to perform the home-based exercises correctly and safely. During the second supervised session, patients are familiarised with the exercises of the supervised RT protocol and the one repetition maximum (1-RM) will be assessed for each exercise. If the kinesiologist considers that a reliable 1-RM assessment cannot be performed in a participant, the participant is instructed to perform the exercises based on perceived exertion using the 6–20 Borg rating of perceived exertion scale.^21^ Patients are familiarised with the scale before enrolment to RT. All participants are instructed to train at a Borg score between 12 and 14, corresponding to ‘somewhat hard’. During the following sessions, the prescribed training scheme (online supplemental table 2B) serves as a basis, progressing from three sets of 10–14 repetitions at 60% of the 1-RM to three sets of 8–10 repetitions at 75% of 1-RM. Adjustments to this scheme can be made according to the Borg scores to ensure that participants consistently train within the target range of Borg scores 12–14. Each set of exercises is performed with a lifting cadence of 1 s concentric and 1 s eccentric contraction with 60 s of rest between each set.^22^ During the 12th session, the 1-RM will be reassessed to prescribe RT for the remaining sessions.

### Protein intake

#### Standard care

As part of the multidisciplinary standard care, patients receive dietary guidance from dieticians. All patients are provided with examples of a balanced daily dietary intake to meet all essential macronutrient and micronutrient requirements.

#### Intervention: protein supplementation

3 weeks post-MBS, patients will start with consumption of 20 g of whey protein once a day for 4 weeks, followed by 20 g of whey protein two times per day for the next 4 weeks and finally 20 g of whey protein three times a day for the last 4 weeks (figure 1). The whey protein supplement (Nutri Whey ProHeat, Friesland Campina, Amersfoort, the Netherlands) is a heat-stable whey protein derived from cheese whey which is concentrated to an 83% protein content. The protein supplement is provided as a powder which is soluble in fluids. To support adherence and manage tolerability, counselling sessions with a dietician are scheduled every 3–4 weeks during this phase.

#### Intervention: dietary protein intake

In the second phase, the focus for the dietary protein intake shifts from protein supplementation to enhancing dietary protein intake of minimally 60 g/day. To support and promote a long-lasting incorporation of sufficient protein intake into the daily dietary intake pattern, additional counselling sessions with a dietician will be scheduled every 2–4 weeks during this phase. Additionally, participants will be provided with examples of dietary adjustments. A total of six counselling sessions with a dietician will be provided to support patients with translating these changes into their daily routine.

### Follow-up

After the 6-month intervention period, all participants in the intervention group will continue with the standard care programme. During the 6-month follow-up period, no additional counselling sessions with the dieticians, nor supervised RT sessions will be provided.

### Outcome measures

The primary outcome is the difference in proportion of patients with disproportional FFML/TWL (ie, ≥30%) in the intervention group compared with the control group at 3 months post-MBS. Secondary outcomes are postintervention differences in body composition, muscle strength and function, cardiorespiratory fitness, (cardio)metabolic health, HRQoL, gastrointestinal discomfort, cost-effectiveness of the intervention and treatment satisfaction in the intervention group. Most outcomes are measured preoperatively and at 3, 6 and 12 months post-MBS. Cost data are assessed preoperatively and at 6 and 12 months post MBS, and treatment satisfaction is assessed after the 6-month intervention period. Cost and outcome data are used to calculate the incremental cost-effectiveness of the intervention in terms of cost per successfully treated patient using a time horizon of 1 year.

The primary outcome was set at 3 months post-MBS as previous studies have shown that the most pronounced changes in body composition, including substantial reduction in FFM, occur during this early postoperative phase.^5 23^ Our intervention, combining RT and protein supplementation, was specifically designed to target this critical period. Secondary outcomes will be assessed at 6 and 12 months to assess the persistence of potential effects over time.

### Measurements

#### Anthropometrics and body composition

Body height and weight will be measured during each measurement visit, using a stadiometer (BSM 170B, InBody, Seoul, South Korea) and will be reported to the nearest 1 mm. Body weight (kg) will be measured using a bioelectrical impedance machine (InBody 770, Seoul, South Korea).^24^ Both measurements will be used to calculate BMI (kg/m^2^). Waist circumference (cm) will be measured midway between the lower rib margin and iliac crest.

Body composition will be assessed in the entire study population by bioelectrical impedance analysis (BIA) (InBody 770, Seoul, South Korea), which can distinguish between FM and FFM. BIA is a validated, low-cost and readily available technique for assessing body composition changes in longitudinal study designs.^25^ Weight loss, FM loss and FFML between preoperative and follow-up assessments will be determined, and the FFML/TWL will be calculated as the absolute FFML (kg) divided by TWL (kg) × 100%.

DXA measurements (Hologic Horizon A, Marlborough, Massachusetts, USA) will be performed in the subgroup (n=100) before and 3 months after surgery, as this is considered the gold standard for measuring body composition. Within the same subgroup, an additional BIA measurement using a different device (TANITA DC-360, Amsterdam, the Netherlands) will be conducted to validate the different methods of body composition assessment.

#### Muscle strength and physical performance

Maximum strength (kg) will be assessed to the nearest 0.1 kg using a leg extension machine (Leg Extension Rev, Technogym, Cesena, Italy) by performing a single repetition isometric strength test. Additionally, a six-repetition maximum power (Watt) test will be performed to assess maximum power to the nearest 0.1 Watt and to assess maximum power acceleration (deg/s) to the nearest 0.1 deg/s.

Maximal handgrip strength (kg) will be assessed to the nearest 0.1 kg using a hand size adjusted dynamometer (InGrip Hand Dynamometer, InBody, Seoul, South Korea). The measurement will be conducted alternately with both arms, twice for each arm, with ±10 s rest between measurements. The maximum strength is defined as the maximum value (kg) of the two measurements for each arm.

The Short Physical Performance Battery (ie, a short balance, walking and chair-rise test) will be performed to determine functional capacity.^26^

In the subgroup (n=100), habitual PA patterns will be assessed using ActivPAL (ActivPAL3 micro, PAL Technologies, Glasgow, UK),^27^ a small device (25×45×5 mm) attached to the participant’s thigh using hypoallergenic tape and sealed for waterproof protection. ActivPAL combines a triaxial accelerometer with an inclinometer which can accurately distinguish between sitting, standing and walking.^28^ Participants will be instructed to wear the ActivPAL for 24 hours/day for 8 consecutive days following each measurement visit. Raw ActivPAL data will be extracted using PAL Software Suite V.9 (Pal Technologies, Glasgow, UK) and analysed using the ActiPASS data processing software^29^ to determine time spent in light PA (ie, metabolic equivalent of a task (MET)-value <3), and moderate-to-vigorous PA (ie, MET-value ≥3).^27 30^

#### Cardiorespiratory fitness

Cardiorespiratory fitness (defined as maximum oxygen uptake (VO_2_ max)) will be assessed using the Åstrand test, a 6 min submaximal cycle ergometer test, which is preceded by 2 min of warming-up. During this test, the participant will be instructed to aim for 50–60 rotations per minute while the resistance of the cycle ergometer will be increased every 30 s until a heart rate (HR) of ∼120 bpm is reached. Resistance will be increased a maximum of four times within the first 2 min of the test. When the aimed HR is reached, the participant will be instructed to continue cycling at this resistance level for the remainder of the test. The VO_2_ max will be estimated by combining the achieved maximum output and HR from the last minute of the test.

#### Blood sampling

Fasted venous blood samples will be drawn from an antecubital vein preoperative, and 3, 6 and 12 months postoperative, to provide information on fasting glucose and insulin, glycated haemoglobin, lipid profile and high sensitive C reactive protein (hs-CRP). The blood will be collected in serum tubes (serum separator tubes, SSTII advance, BD Vacutainer, Becton Dickinson, Franklin Lakes, New Jersey, USA), lithium-heparin plasma tubes (plasma separator tubes, LH PSTII, BD Vacutainer) and EDTA tubes (K2E, BD Vacutainer). The serum tubes and the lithium-heparin plasma tubes will be centrifuged according to the manufacturer’s instructions. From the EDTA tubes, whole blood samples will be collected. Aliquots will initially be stored at −20°C at the research sites and will be transferred within 4 weeks to the Radboudumc for long-term storage at −80°C.

#### Blood pressure

Following ≥5 min of seated rest, three consecutive blood pressure measurements will be performed using an automatic cuff (Omron, Kyoto, Japan), with a 1 min interval between measurements. The last two measures will be averaged to obtain mean HR, mean systolic blood pressure and mean diastolic blood pressure.

#### Protocol adherence

For participants in the intervention group, adherence to the RT protocol will be monitored via the Technogym application (Technogym, Cesena, Italy), which allows participants to log both completion of the home-based and supervised RT sessions. Additionally, adherence to the home-based RT protocol will be assessed using an online single question survey (Castor EDC, Castor). Attendance at the supervised RT sessions will be recorded using an attendance list. Adherence to the whey protein supplementation will be assessed using a brief weekly online survey (Castor EDC, Castor).

### Questionnaires

After each measurement visit (ie, baseline, 3, 6 and 12 months postoperatively), participants will receive an email with a personal weblink to an online questionnaire (Castor EDC, Castor) to assess HRQoL and gastrointestinal symptoms and a separate weblink to an online Food Frequency Questionnaire (FFQ tool, Wageningen University and Research, Wageningen, The Netherlands). In addition to the online questionnaires, a brief set of structured questions will be asked in person during each measurement visit. These questions address current engagement in RT (eg, type of exercise and frequency), weekly performance of light, moderate and vigorous PA and prior use of glucagon-like peptide 1 receptor agonists for weight loss.

#### Health-related quality of life

HRQoL will be evaluated using the OBESI-Q, a translated subset of the BODY-Q questionnaire, a validated patient-reported outcome instrument that is especially designed to measure a patient’s perception on weight loss and/or body contouring.^31^ The OBESI-Q questionnaire is designed to evaluate HRQoL in patients with obesity through 54-point Likert-scale (*Completely disagree* to *Completely agree* or *Never* to *Always*) questions, divided over five subscales: *eating behaviour, social function, psychological function, physical function* and *body image*. For each subscale, a sum score will be calculated. As part of the standard perioperative care at the NOK, patients are asked to fill in the OBESI-Q at baseline and after 12 months of follow-up. These data will be extracted from the electronic health record. A shortened version of the OBESI-Q will be sent from Castor EDC at 3 and 6 months postoperatively, including the subscales eating behaviour, physical function and body image, as we anticipate observing the most pronounced changes within these domains in response to our intervention.

In addition to the OBESI-Q, the five-level EuroQol five-dimensions (EQ-5D-5L) questionnaire will be administered orally during each measurement visit to assess HRQoL. The EQ-5D-5L is a validated instrument suitable for use in cost-effectiveness analyses.^32 33^

#### Gastrointestinal tolerability

Gastrointestinal tolerability of the protein supplementation will be assessed using the Gastrointestinal Symptoms Rating Scale (GSRS).^34^ The GRSR is a validated questionnaire, consisting of 15 questions on a 7-point Likert scale (*no discomfort at all* to *very severe discomfort*), divided over five subscales: *reflux, abdominal pain, indigestion, diarrhoea* and *constipation*. For each subscale, a sum score will be calculated.

#### Habitual dietary intake

Habitual dietary intake will be assessed using an online FFQ (FQ13, Wageningen University & Research), a validated questionnaire to assess nutrient intake.^35 36^ This information will be used to calculate total energy intake and intake of macronutrients and micronutrients.

### Data management

All data will be stored in a digital research environment (myDRE, anDREa, Nijmegen, The Netherlands) for at least 15 years, adhering to the General Data Protection Regulation, Good Clinical Practice and Good Laboratory Practice guidelines. Standard software packages will be used for data handling and statistical analyses (eg, Castor EDC, IBM SPSS, R, Microsoft Excel). A comprehensive data catalogue is presented in table 2. In line with open science and the findable, accessible, interoperable and reusable (FAIR) principles, inquiries for reuse of the ENRICHED data can be directed to the corresponding author.

**Table 2:** Data catalogue table of the ENRICHED study

Category	Subcategory	Variables
Baseline characteristics	Study inclusion	Allergic or sensitive to dairy proteins, or lactose intolerant (yes/no); diagnosed renal insufficiency (yes/no); diagnosed intestinal disease influencing uptake of protein (yes/no); inability to perform any resistance exercises (yes/no); previous MBS (yes/no); presence of medical devices which are contraindicated for BIA (yes/no); age >18 (yes/no); MBS scheduled (yes/no); able to understand and perform study procedures (yes/no); inclusion criteria met (yes/no); informed consent signed (yes/no); date of informed consent (dd-mm-yyyy).
	Demographics	Year of birth (yyyy); sex (female/male); medical history (text); Charlson Comorbidity Index (none/myocardial infarction/congestive heart failure/peripheral vascular disease/cerebrovascular disease (CVA or TIA)/dementia/chronic pulmonary disease/peptic ulcer disease/mild liver disease/hemiplegia/moderate-to-severe chronic kidney disease/tumour without metastasis/tumour with metastasis/leukaemia/lymphoma/HIV or AIDS/connective tissue disease/diabetes without organ damage/diabetes with organ damage/other (text).
	Randomisation	Intervention or control group (intervention group/control group); subgroup (yes/no).
Surgery	Surgery characteristics	Date of surgery (dd-mm-yyyy); type of surgery (RYGB/SG).
Measurements	Visit information	Date of visit (dd-mm-yyyy).
	Exercise characteristics	Do you usually do muscle-strengthening exercises (yes/no); if yes: how many days, in a usual week, do you do muscle-strengthening exercise (0/1/2/3/4/5/6/7); and: which type of muscle-strengthening exercise do you do (holistic exercises/body weight exercises/resistance band or free weights/weight machines)?
	Regular physical activity	Do you carry out light/moderate/vigorous physical activity on a regular basis (yes/no); if yes: how many days a week do you carry out light/moderate/vigorous physical activity (days/week); and: on those days, how many min a day do you do light/moderate/vigorous physical activity (min/day)?
	Previous GLP-1 use	Have you previously used GLP-1 medication (yes/no); if yes: which type of GLP-1 medication have you used (liraglutide (Saxenda)/semaglutide (Ozempic/Wegovy)/tirzepatide (Mounjaro)/I do not know); and: for how long did you use this medication (months); and: how much weight did you lose while using this medication (kg)?
	Healthcare consumption	In the past 3 months, have you had one or more appointments with any of the following healthcare providers (general practitioner/medical specialist/paramedical healthcare provider (please specify)/none of the above); if yes: how many appointments have you had in the past 3 months; in the past 3 months, have you visited a hospital or another type of healthcare facility (no/yes, a hospital/yes, another type of healthcare facility (please specify); if yes: what was the purpose of your visit (day treatment/inpatient admission/outpatient consultation); if ‘inpatient admission’: how many days were you admitted to the hospital or healthcare facility (days); did you undergo a medical procedure during your visit (yes/no)?
	Patient-reported health	Mobility (I have no/slight/moderate/severe problems walking or I am unable to walk); ability to wash or dress yourself (I have no/slight/moderate/severe problems washing or dressing myself or I am unable to wash or dress myself); ability to perform your daily activities (I have no/slight/moderate/severe problems performing my daily activities or I am unable to perform my daily activities); level of pain or discomfort (I have no/slight/moderate/severe/extreme pain or discomfort); emotional state (I am not/slightly/moderately/severely/extremely anxious or depressed).
	Multivitamins	Use of (multi)vitamins (no multivitamins/Fit For Me (RYGB)/Elan (RYGB)/vitamins on prescription (RYGB)/Flinndal (RYGB)/Lucovitaal WLS (RYGB)/Fit For Me (SG)/Elan (SG)/vitamins on prescription (SG)/Flinndal (SG)/house brand vitamins/other (text); if not ‘No multivitamins’; dosage (multi)vitamins (two tablets per day/one tablet per day/five to six tablets per week/three to four tablets per week/one to two tablets per week). Calcium and vitamin D supplement use (no calcium and vitD/500 mg calcium and 400IE vitD/500 mg calcium and 800IE vitD/1000 mg calcium and 800IE vitD/Other (text); if not ‘No calcium and vitD’; dosage calcium and vitD supplements (four tablets per day/three tablets per day/two tablets per day/one tablet per day/five to six tablets per week/three to four tablets per week/one to two tablets per week).
	Body composition	Height (cm); weight (kg); BMI (kg/m^2^); waist circumference (cm); fat mass (kg); fat mass (%); fat-free mass (kg); fat-free mass (%); fat-free mass right arm (kg); fat-free mass left arm (kg); fat-free mass trunk (kg); fat-free mass right leg (kg); fat-free mass left leg (kg).
	Physical tests	Handgrip measurements: handgrip right hand first measurement (kg); handgrip right hand second measurement (kg); handgrip left hand first measurement (kg); handgrip left hand second measurement (kg). Leg extension measurement: bilateral maximum isometric strength (kg); bilateral maximum power (Watt); bilateral maximum power acceleration (deg/s). SPPB: balance feet together during 10 s (yes/no); if yes: balance semitandem during 10 s (yes/no); if yes: balance full tandem (seconds); time 4 m walk (seconds); time chair stand (seconds); SPPB score (scale 0–12). Åstrand ergometer test: use of beta blockers (yes/no); if no: wattage at finish (Watt); mean heart rate 5th and 6th min (bpm); Borg RPE scale at finish (scale 6–20); predicted VO^2^ max (absolute VO^2^ max) (L/min); predicted VO^2^ max per kg (relative VO^2^ max) (L/min/kg).
	Blood pressure	Heart rate at rest (bpm); systolic blood pressure at rest (mm Hg); diastolic blood pressure at rest (mm Hg).
	Blood sample	Blood sample drawn (yes/no); if yes: date of blood sample drawn (dd-mm-yyyy); if no: reason no blood drawn (text).

BIA, bioelectrical impedance analysis; BMI, body mass index; bpm, beats per minute; cm, centimetres; CVA, cerebrovascular accident; ENRICHED, EffectiveNess of pRotein supplementatIon Combined witH resistance Exercise training to counteract Disproportional fat-free mass loss following metabolic–bariatric surgery; GLP-1, glucagon-like peptide 1; kg, kilograms; L, litres; MBS, metabolic–bariatric surgery; min, minutes; RPE, rating of perceived exertion; RYGB, Roux-en-Y gastric bypass; SG, sleeve gastrectomy; SPPB, Short Physical Performance Battery; TIA, transient ischaemic attack; vit, vitamin; VO_2_ Max, maximum uptake of oxygen; WLS, weight loss surgery.

### Sample size

Sample size calculation is based on the primary outcome of FFML/TWL ≥30% at 3 months post-MBS. The power analysis was based on differences between two independent groups (intervention group vs control group). We consider our intervention successful if we find an expected difference in prevalence of disproportional FFML of 26%^5^ versus 14% between the control and intervention group, respectively. Based on a two-sided test with α=0.05 and a power of 80%, 174 participants per study arm are needed to show this difference. Accounting for a 10% drop-out rate, a total of 400 participants will be recruited (200 per study arm). Calculations were performed using G-power V.3.1.2. (University of Düsseldorf, Düsseldorf, Germany)

### Statistical analysis

Statistical analyses will be performed using R. Collected data will be checked for completeness and normality. Continuous parametric data will be displayed as mean±SD, non-parametric data as median (IQR) and categorical data as frequency with percentage. All statistical tests will be two-sided, and significance is set at p<0.05.

Descriptive analyses will be performed to explore the variance in the study parameters and calculate the baseline characteristics of the study population. For the primary analysis, an intention-to-treat approach will be used. Generalised estimating equations will be used for dichotomous outcomes grouped by patient ID to account for repeated measurements, including an adjustment for NOK centre and, if possible, adjusted for the baseline value of the outcome variable. A similar approach will be used for continuous outcomes using linear mixed-models analyses with a random intercept for patient ID to account for repeated measurements. The intervention effects will be evaluated for all follow-up time points by including a dummy variable for each time point and repeating the analyses with a different reference time point. Potential effect modification will be evaluated for sex, age group (age <50 years and age ≥50 years), type of surgery, study site and postmenopausal status (in female participants). A variable is treated as an effect modifier when its interaction term with the independent variable has a p value <0.10. When effect modification is observed, stratified results will be reported in addition to the overall results. Additionally, confounding will be evaluated for sex, age, baseline BMI, treatment adherence and type of surgery by including each variable in the model separately. Sensitivity analyses will be conducted to assess the robustness of the findings.

### Cost-effectiveness

As part of the ENRICHED trial, a cost-effectiveness analysis will be performed, focusing on formal care costs over a period of 12 months. These costs will include routine costs of standard perioperative care, intervention costs (eg, protein supplementation, RT equipment and personnel) and general healthcare consumption costs. Healthcare consumption will be assessed using extracted data from the electronic patient records, supplemented by selected items from the iMTA Medical Consumption Questionnaire.^37^ Additionally, HRQoL will be assessed using the EQ-5D-5L questionnaire.^32^ The incremental costs between the intervention and standard care will be related to the difference in proportion of patients achieving the success criterion defined as FFML/TWL ≥30% at 12 months.

### Patient and public involvement

Patients and healthcare professionals were involved in the design of the ENRICHED protocol and the practical implementation of the intervention in the NOK clinics through focus group interviews. Two separate focus groups were held during the design phase of the study protocol. For these group sessions, patients and healthcare professionals (eg, dieticians, kinesiologists, psychologists and bariatric physicians) were invited to participate in a semistructured group discussion, aimed at gathering input on the feasibility of the intervention and to identify any potential challenges and barriers. The insights gained from these sessions were incorporated into the development of the study protocol. This approach to engage relevant stakeholders aimed to ensure that the intervention is not only evidence-based but also tailored to meet the practical needs in a real-world clinical setting.

## Ethics and dissemination

This study protocol has been developed in accordance with the Standard Protocol Items: Recommendations for Interventional Trials 2025 statement^38^ and has been reviewed and approved by the Medical Research Ethics Committee Oost-Nederland (NL-OMON57119). Any substantial amendments to the protocol will be submitted for ethical approval prior to implementation. Written informed consent will be obtained from all participants prior to enrolment (see online supplemental material for the informed consent form). Participants will be informed of the possibility to withdraw from the study at any time without consequences. The results of the ENRICHED trial will be disseminated in line with the Consolidated Standards of Reporting Trials 2025 statement^39^ through peer-reviewed scientific journals and presentations at national and international conferences. Data will be shared on reasonable request after publication of the main trial results.

### Monitoring

Given the negligible risk associated with this study, no formal data monitoring committee has been established. However, an independent monitor has been appointed to verify the informed consent procedure shortly after study initiation and conduct interim on-site visits to review data entry and reported serious adverse events and suspected unexpected serious adverse reactions. On study completion, the monitor will perform a close-out visit to ensure proper closure and documentation.

## Discussion

While MBS is highly effective for achieving substantial and sustained weight loss, the need to preserve FFM post-MBS is often overlooked. Disproportionate FFML has been associated with impaired metabolic function, reduced physical capacity and increased risk of chronic diseases such as type 2 diabetes and cardiovascular diseases.^4^,^69 40^ However, only a few studies have evaluated strategies to mitigate FFML in the early postoperative phase following MBS. The ENRICHED trial is designed to address these gaps by evaluating the effectiveness of an intervention combining RT and whey protein supplementation on FFML, body composition, muscle strength and function, cardiorespiratory fitness, (cardio)metabolic health, HRQoL, gastrointestinal discomfort and cost-effectiveness of the intervention.

The intervention includes both supervised and home-based RT sessions, tailored to the participants’ capacity and progression. The use of the Technogym application introduces real-time tracking of training frequency and intensity. In addition, habitual PA will be objectively assessed using accelerometry in a subgroup, enabling detailed analysis of daily activity patterns and potential dose–response relationships.

Previous studies investigating RT and protein supplementation post-MBS have shown mixed results, often limited by a small or specific sample size, or mid to long-term follow-up only.^17^,^19^ For example, one RCT reported improved muscle strength in the group receiving RT and protein supplementation, but no effect on lean body mass at 6 months post-MBS. This trial was limited by a relatively small sample size (n=76), and a homogeneous study population (eg, exclusively women undergoing RYGB), limiting the generalisability of the findings.^17^ Another study did find a positive association between a combined intervention of RT and protein supplementation and increased FFM and skeletal muscle mass, but this study focused on the long-term follow-up (2–7 years), rather than the early postoperative period when FFML is most pronounced.^19^ In other studies, focusing exclusively on RT or protein supplementation post-MBS, the absence of a combined intervention limits the ability to assess potential synergistic effects on FFM preservation.^1641^,^43^ The ENRICHED trial addresses these limitations by including a larger and more general population (ie, both men and women, undergoing either RYGB or SG), and by focusing on the early postoperative period.

Limitations observed in previous studies have been low compliance to protein intake protocols, potentially due to gastrointestinal discomfort and lacking information on habitual PA patterns.^43^ The protein supplementation protocol in the ENRICHED trial is based on recent findings that consumption of whey protein resulted in the highest increase in overall daily protein intake, compared with other types of protein supplementation strategies.^44^ Additionally, whey protein was found superior to other types of protein, such as soy, casein or hydrolysed collagen proteins, to increase muscle protein synthesis.^45^ Adherence to the ENRICHED protocol is closely monitored, enhancing the interpretation of the true effect of the intervention. Moreover, the intervention is embedded in the standard perioperative care programme.

In addition to clinical outcomes, the ENRICHED trial includes a cost-effectiveness analysis to evaluate the economic impact of the intervention. By combining quality-adjusted life years (QALYs) with data on healthcare consumption costs, this analysis will provide valuable insights into the potential for the implementation of the intervention in routine care.

The present study could have several strengths, including its multicentre design, large sample size and implementation of the intervention into a routine clinical setting. However, some limitations should be acknowledged. First, blinding of participants and care providers is not possible due to the nature of the intervention, which may introduce performance bias. Second, although DXA measurements are considered the gold standard for body composition assessment, it is not always feasible for repeated use due to cost, limited availability and radiation exposure. BIA, although validated, may be less precise. Finally, adherence to both RT and protein intake may vary, despite monitoring and counselling, which may influence outcomes.

The ENRICHED trial will implement an innovative protocol combining home-based and supervised RT complemented with whey protein supplementation to improve postoperative outcomes in patients undergoing MBS. Application of evidence-based dietary and RT strategies with accurate monitoring in a real-world clinical setting can potentially contribute to revision of standard clinical care guidelines for a patient-tailored intervention for the preservation of FFM.

## Supplementary material

10.1136/bmjopen-2025-108346 online supplemental file 1

10.1136/bmjopen-2025-108346 online supplemental file 2

10.1136/bmjopen-2025-108346 online supplemental file 3
