# Supplementary material for: Effectiveness of protein supplementation combined with resistance training to counteract disproportional fat-free mass loss following metabolic bariatric surgery: rationale and design of the ENRICHED randomised controlled trial
Source: BMJ Open. 2025 Dec 29;15(12):e108346. doi: 10.1136/bmjopen-2025-108346 (PMC12750799; doi:10.1136/bmjopen-2025-108346)
Supplement: online supplemental file 1 [file bmjopen-15-12-s001.docx]

**SUPPLEMENTAL TABLE 1**

| **Supplemental Table 1. Home-based resistance training protocol** | | | |
| --- | --- | --- | --- |
| **Level 1** | | | |
| **Exercise** | **Description** | **Sets x Reps** | **Rest** |
| 1. Wall push-up | Stand facing a wall, feet shoulder-width apart. Place the hands on the wall, and bend the elbows to bring the chest towards the wall, then push back to the start. | 4 x 8-12 reps | 60 seconds between sets |
| 2. Half squat | Stand with the feet shoulder-width apart. Bend the knees to ~45°, then return to standing. | 4 x 8-12 reps | 60 seconds between sets |
| 3. Inclined plank | Place the hands on an elevated surface (e.g., chair, table or block), feet shoulder-width apart. Hold the plank position for 20 seconds. | 4 x 20 seconds | 60 seconds between sets |
| 4. Glute bridge | Lie on the back, feet flat on the floor. Engage the core and glutes, and lift the hips to form a straight line from shoulders to knees, then lower slowly and return to the start. | 4 x 8-12 reps | 60 seconds between sets |
| 5. Arm lifts | Stand upright, arms beside the body. Raise the arms sideways to shoulder height, then lower slowly back to the start. | 4 x 8-12 reps | 60 seconds between sets |
| 6. Forward bend with staggered stance | Stand with one foot in front of the other. Bend forward at the hips until the back and legs form a 90° angle, then return to standing. | 4 x 8-12 reps | 60 seconds between sets |
| **Level 2** | | | |
| **Exercise** | **Description** | **Sets x Reps** | **Rest** |
| 1. Inclined push-up | Place the hands on an elevated surface (e.g., chair, table or block), feet shoulder-width apart. Bend the elbows to bring the chest towards the surface, then push back to the start. | 4 x 8-12 reps | 60 seconds between sets |
| 2. Chair squat | Stand in front of a chair, feet shoulder-width apart. Bend the knees to until the buttocks touch the chair, then return to standing. | 4 x 8-12 reps | 60 seconds between sets |
| 3. Forearm plank on the knees | Place the forearms and knees on the floor, both shoulder-width apart. Hold the plank position for 20 seconds. | 4 x 20 seconds | 60 seconds between sets |
| 4. Glute bridge | Lie on the back, feet flat on the floor. Engage the core and glutes, and lift the hips to form a straight line from shoulders to knees, then lower slowly and return to the start. | 4 x 8-12 reps | 60 seconds between sets |
| 5. Weighted arm lifts | Stand upright, arms beside the body, holding a weight in both hands. Raise the arms sideways to shoulder height, then lower slowly back to the start. | 4 x 8-12 reps | 60 seconds between sets |
| 6. Forward bend with parallel stance | Stand with feet shoulder-width apart. Bend forward at the hips until the back and legs form a 90° angle, then return to standing. | 4 x 8-12 reps | 60 seconds between sets |
| **Level 3** | | | |
| **Exercise** | **Description** | **Sets x Reps** | **Rest** |
| 1. Push-up on the knees | Place the hands and knees on the floor, both shoulder-width apart. Bend the elbows to bring the chest towards the floor, then push back to the start. | 4 x 8-12 reps | 60 seconds between sets |
| 2. Squat with bodyweight | Stand with feet shoulder-width apart. Bend the knees to a 90° angle, then return to standing. | 4 x 8-12 reps | 60 seconds between sets |
| 3. Forearm plank on the toes | Place the forearms and toes on the floor, both shoulder-width apart. Hold the plank position for 20 seconds. | 4 x 20 seconds | 60 seconds between sets |
| 4. Single-leg glute bridge | Lie on the back, feet flat on the floor. Engage the core and glutes, and lift the hips to form a straight line from shoulders to knees while extending one leg, then lower slowly. | 4 x 8-12 reps | 60 seconds between sets |
| 5. Weighted arm lifts | Stand upright, arms beside the body, holding a weight in both hands. Raise the arms sideways to shoulder height, hold this position for 5 seconds, then lower slowly back to the start. | 4 x 8-12 reps | 60 seconds between sets |
| 6. Single-leg forward bend | Stand on one leg. Bend forward at the hips until the back and legs form a 90° angle, then return to standing. | 4 x 8-12 reps | 60 seconds between sets |
| **Level 4** | | | |
| **Exercise** | **Description** | **Sets x Reps** | **Rest** |
| 1. Full push-up | Place the hands and feet on the floor, both shoulder-width apart. Bend the elbows to bring the chest towards the floor, then push back to the start. | 4 x 8-12 reps | 60 seconds between sets |
| 2. Weighted squat | Stand with feet shoulder-width apart, holding a weight at the chest. Bend the knees to a 90° angle, then return to standing. | 4 x 8-12 reps | 60 seconds between sets |
| 3. Forearm plank on the toes | Place the forearms on the floor, feet shoulder-width apart. Hold the plank position for 30 seconds. | 4 x 30 seconds | 60 seconds between sets |
| 4. Single-leg glute bridge | Lie on the back, feet flat on the floor. Engage the core and glutes, and lift the hips to form a straight line from shoulders to knees while extending one leg, hold this position for 5 seconds, then lower slowly. | 4 x 8-12 reps | 60 seconds between sets |
| 5. Weighted arm lifts | Stand upright, arms beside the body, holding a weight in both hands. Raise the arms sideways to shoulder height, hold this position for 5 seconds, then lower slowly back to the start. | 4 x 8-12 reps | 60 seconds between sets |
| 6. Weighted single-leg forward bend | Stand on one leg holding a weight in both hands. Bend forward at the hips until the back and legs form a 90° angle, then return to standing. | 4 x 8-12 reps | 60 seconds between sets |
