# Supplementary material for: Effectiveness of protein supplementation combined with resistance training to counteract disproportional fat-free mass loss following metabolic bariatric surgery: rationale and design of the ENRICHED randomised controlled trial
Source: BMJ Open. 2025 Dec 29;15(12):e108346. doi: 10.1136/bmjopen-2025-108346 (PMC12750799; doi:10.1136/bmjopen-2025-108346)
Supplement: online supplemental file 2 [file bmjopen-15-12-s002.docx]

**SUPPLEMENTAL TABLE 2**

| **Supplemental Table 2A. Supervised resistance training protocol** | | |
| --- | --- | --- |
| **Exercise** | **Equipment** | **Description** |
| 1. Leg extension | Leg extension machine | Seated with the back against the support, extend the legs until nearly straight, then return to the starting position. |
| 2. Lat pull down | Lat pull down machine | Pull the bar towards the chest while seated upright. Hold this position briefly, then extend the arms to return to the starting position. |
| 3. Cable hip extension | Cable tower machine | Stand with the back towards the machine, pull the cable forward by extending the hips until the torso is upright, then return to the starting position. |
| 4. Push up | - | Place the hands and feet or knees on the floor, both shoulder-width apart. Bend the elbows to bring the chest towards the floor, then push back to the starting position. |
| 5. Upright row | Cable tower machine | Stand upright, holding the cable at hip height. Pull the cable to the chest with the elbows to the sides, then return to the starting position. |

| **Supplemental Table 2B. Supervised resistance training progression schedule** | | | | | | |
| --- | --- | --- | --- | --- | --- | --- |
| **Session** | **Sets x repetitions** | **Intensity (%1RM)** |  | **Session** | **Sets x repetitions** | **Intensity (%1RM)** |
| 1 | Explanation of home-based RT exercises | |  | 13 | 3 x 10-14 | 60% |
| 2 | Assessment of 1RM | |  | 14 | 3 x 15-18 | 60% |
| 3 | 3 x 10-14 | 60% |  | 15 | 3 x 10-12 | 65% |
| 4 | 3 x 15-18 | 60% |  | 16 | 3 x 13-15 | 65% |
| 5 | 3 x 10-12 | 65% |  | 17 | 3 x 9-11 | 67.5% |
| 6 | 3 x 13-15 | 65% |  | 18 | 3 x 13-15 | 67.5% |
| 7 | 3 x 9-11 | 67.5% |  | 19 | 3 x 6-8 | 70% |
| 8 | 3 x 9-11 | 70% |  | 20 | 3 x 9-11 | 70% |
| 9 | 3 x 6-8 | 72.5% |  | 21 | 3 x 6-8 | 72.5% |
| 10 | 3 x 5-7 | 75% |  | 22 | 3 x 6-8 | 75% |
| 11 | 3 x 8-10 | 75% |  | 23 | 3 x 8-10 | 75% |
| 12 | Reassessment of 1RM | |  | 24 | 3 x 10-14 | 75% |
| 1RM, one-repetition maximum; RT, resistance training | | | | | | |
