# Supplementary material for: Effectiveness of protein supplementation combined with resistance training to counteract disproportional fat-free mass loss following metabolic bariatric surgery: rationale and design of the ENRICHED randomised controlled trial
Source: BMJ Open. 2025 Dec 29;15(12):e108346. doi: 10.1136/bmjopen-2025-108346 (PMC12750799; doi:10.1136/bmjopen-2025-108346)
Supplement: online supplemental file 3 [file bmjopen-15-12-s003.pdf]

Radboudumc

Informed Consent Form ENRICHED

- I have read the information letter. I have been able to ask questions, and my questions have been answered adequately. I have had sufficient time to decide whether I want to participate.
- I understand that participation is voluntary. I understand that I may decide at any time not to participate in the study or to withdraw from participation. I do not need to give a reason for withdrawing.
- I give the researcher permission to inform my medical specialist that I am participating in this study.
- I give the researcher permission to request information from my medical specialist regarding my medical treatment.
- I give the researchers permission to collect and use my data and biological material. The researchers will only use these to answer the research question of this study.
- I understand that, in the context of the study, some people have access to all my data. These people are listed in the information letter. I give these people permission to review my data for this purpose.

Please select ‘Yes’ or ‘No’ in the table below:

I give permission to store my data and use it for other research, as described in the information letter	☐ Yes<br>☐ No
I give permission to store my (remaining) biological material to use it for other research, as described in the information letter. The biological material will be stored for 15 years.	☐ Yes<br>☐ No
I give permission to be contacted again after this study for follow-up research.	☐ Yes<br>☐ No

Participant’s name...................... Date __/__/__

Signature.......................

I declare that I have fully informed this participant about the study described above.

If any new information becomes available during the study that may affect the participant’s consent, I will inform the participant in a timely manner.

Researcher’s name (or representative) .... Date __/__/__

Signature.......................
