# Supplementary material for: Effectiveness of protein supplementation combined with resistance training to counteract disproportional fat-free mass loss following metabolic bariatric surgery: rationale and design of the ENRICHED randomised controlled trial
Source: BMJ Open. 2025 Dec 29;15(12):e108346. doi: 10.1136/bmjopen-2025-108346 (PMC12750799; doi:10.1136/bmjopen-2025-108346)
Supplement: Supplementary file 4 [file bmjopen-2025-108346.reviewer_comments.pdf]

PEER REVIEW HISTORY

BMJ Open publishes all reviews undertaken for accepted manuscripts. Reviewers are asked to complete a checklist review form and are provided with free text boxes to elaborate on their assessment. These free text comments are reproduced below.

ARTICLE DETAILS

Title (Provisional)

Effectiveness of protein supplementation combined with resistance training to counteract disproportional fat-free mass loss following metabolic bariatric surgery: rationale and design of the ENRICHED randomised controlled trial

Authors

de Roos, Bente M.; Yeh, Lin-Lin Yessica; van den Hooff, Puck S.; Nuijten, Malou A.H.; Twisk, Jos W.R.; Liem, Ronald; van Acker, Gijs J.D.; Severens, Johan L.; Kambic, Tim; Serlie, Mireille; Monpellier, Valerie; Eijsvogels, TMH; Hopman, Maria TE; Karregat, Jan H.M.

VERSION 1 - REVIEW

**Reviewer**	**1**
**Name**	**Welbourn, Richard**
**Affiliation**	**Musgrove Park Hospital, Department of Upper Gastro-intestinal and Bariatric Surgery**
**Date**	**29-Oct-2025**
**COI**	**None**

This protocol is a timely study comparing standard post operative diets after bariatric surgery (MBS) and a protein-enriched diet to see if muscle mass can be preserved better. The endpoints all appear appropriate.

Major points

1 The choice of 3 months for the main endpoint seems rather early, and although patients will be studied up to 12 months, it seems more logical to have the primary endpoint rather later, eg 12 months, and follow up for say 2 years, so that weight loss (and presumably its consequences on fat free mass) will be at their maximum.

2 How easy do the authors expect to find recruitment to the study? Patients knowing that extra protein might be beneficial might prefer to take it anyway if in the control arm, and perhaps would not want to take part in the RCT in any case due to this. What training will the researchers have in equipoise delivery and how will this be incorporated into trial design?

3 It is suggested that the type of surgery should be specified ie Roux en Y gastric bypass, unless the authors have reason to think that the results would be comparable between operations. It would be a purer study for surgery to be standardised.

VERSION 1 - AUTHOR RESPONSE

Reviewer 1 (Comments to the authors)

Comment #1. The choice of 3 months for the main endpoint seems rather early, and although patients will be studied up to 12 months, it seems more logical to have the primary endpoint rather later, eg 12 months, and follow up for say 2 years, so that weight loss (and presumably its consequences on fat free mass) will be at their maximum.

Author response: We thank the reviewer for evaluating our manuscript. We understand the concern that 3 months could be deemed early given the follow-up period up to 12 months postoperatively. The 3-month follow-up was deliberately chosen as the primary endpoint, since previous studies have demonstrated that the most pronounced changes in body composition, including marked reductions in fat-free mass, occur during this early postoperative period. Therefore, we specifically designed our intervention, resistance training complemented with protein supplementation, to target this critical phase.

Furthermore, the intervention period combining both aspects of the intervention lasts 3 months, after which the resistance training continues for an additional 3 months. For this reason, we will also assess outcomes at 6 months as important secondary outcome, and at 12 months to evaluate the sustainability of the effects of the intervention. While we agree that longer-term follow-up could provide valuable insights, our design specifically focuses on the critical period during which significant body composition changes occur.

We acknowledge that the rationale for selecting 3 months as the primary endpoint was not sufficiently substantiated in the original manuscript. Therefore, we have expanded this rationale in the Methods section to clarify why we consider this time point as most relevant.

Copied text from the manuscript:

"The primary outcome was set at three months post-MBS as previous studies have shown that the most pronounced changes in body composition, including substantial reductions in fat-free mass, occur during this early postoperative phase[5,23]. Our intervention, combining RT and protein supplementation, was specifically designed to target this critical period. Secondary outcomes will be assessed at six and twelve months to assess the persistence of potential effects over time." (lines 234-238)

Added reference:

[23] *Hua MB, Li J, Wang TX, et al. Meta-analysis of changes in skeletal muscle mass within 1 year after bariatric surgery. Surg Endosc. Apr 2025;39(4):2668-2676. doi:10.1007/s00464-024-11512-4*

Comment #2. How easy do the authors expect to find recruitment to the study? Patients knowing that extra protein might be beneficial might prefer to take it anyway if in the control arm

and perhaps would not want to take part in the RCT in any case due to this. What training will the researchers have in equipoise delivery and how will this be incorporated into trial design?

<u>Author response:</u> We thank the reviewer for highlighting these important aspects related to the recruitment of participants, potential contamination, and equipoise. Below, we have responded to each aspect individually.

Recruitment feasibility: We are currently recruiting participants in two centers of the NOK, the largest provider of bariatric care in the Netherlands. In each center, a group of ten patients is expected to start each week. We anticipate including 30-40% of these patients, corresponding to approximately six to eight patients per week. Based on this rate, we anticipate achieving our target sample size within the planned recruitment period until December 2026. We have added this anticipated recruitment timeline to the manuscript

Copied text from the manuscript:

"Recruitment of participants began in May 2025 and is expected to continue until December 2026." (line 125)

Reluctance to participate/risk of drop out: We acknowledge the concern that patients may be reluctant to participate in the trial if they believe that being randomized to the control group means refraining from additional protein intake or resistance exercise training. Or that patients in the control group might be less motivated to remain in the study. However, participation in the study does not restrict individuals in the control group from participation to resistance exercise training or consuming additional protein. Moreover, participation offers them valuable insights into their postoperative health and body composition as advanced assessments, including body composition (InBody770) and muscle strength assessment (1RM leg extension) are performed. These benefits are clearly explained to potential participants during the recruitment process. Therefore, we do not expect this to negatively impact the recruitment. We have clarified in our manuscript that participants are informed during recruitment that no restrictions apply to the control group and that both study arms are equally valuable for answering the research question.

Copied text from the manuscript:

"Allocation to our control group does not restrict participants from engaging in resistance training or consuming additional protein. This is emphasized by the research team during recruitment. Moreover, the research team is trained to ensure equipoise delivery during recruitment by presenting both study arms as equally important for the aim of this study." (lines 153-156)

Contamination: we acknowledge the potential for contamination if participants in the control group increase their protein intake or engage in resistance training. To address this, we systematically assess physical activity patterns, engagement in resistance training, and dietary intake at all measurement points in both groups. These data will allow us to adjust for these factors in our statistical analyses.

Comment #3. It is suggested that the type of surgery should be specified ie Roux en Y gastric bypass, unless the authors have reason to think that the results would be comparable between operations. It would be a purer study for surgery to be standardised.

Author response: **We acknowledge that including both Roux-en-Y gastric bypass and sleeve gastrectomy procedures introduce heterogeneity. Yet, previous studies have demonstrated that disproportional FFML is an issue following both types of surgery. Additionally, the indication for surgery type differs between patients. Sleeve gastrectomy is often performed in patients with more severe obesity. Excluding either one of the procedures would limit the generalizability of our findings. We aim to explore surgery type as a potential effect modifier in predefined subgroup analyses. This will allow to investigate whether FFML and the potential effect of the intervention differ between RYGB and SG, which could add to tailored strategies in clinical practice.**

We have added this rationale to the Methods section of our manuscript to substantiate why including both types of surgery is important in our study.

Copied text from the manuscript:

"Both Roux-en-Y gastric bypass and sleeve gastrectomy (SG) procedures are included in this trial to enhance generalizability of our findings and to allow examining whether the intervention is effective across both surgical techniques" (lines 122-124)
